# Diagnostic Ability of Dynamic Contrast-Enhanced Magnetic Resonance Imaging for Prostate Cancer and Clinically Significant Prostate Cancer in Equivocal Lesions: A Systematic Review and Meta-Analysis

**DOI:** 10.3389/fonc.2021.620628

**Published:** 2021-02-19

**Authors:** Jing Zeng, Qingqing Cheng, Dong Zhang, Meng Fan, Changzheng Shi, Liangping Luo

**Affiliations:** ^1^ Medical Imaging Center, The First Affiliated Hospital of Jinan University, Guangzhou, China; ^2^ Engineering Research Center of Medical Imaging Artificial Intelligence for Precision Diagnosis and Treatment, Guangzhou, China

**Keywords:** DCE-MRI, dynamic contrast-enhanced magnetic resonance imaging, prostate cancer, clinically significant prostate cancer, equivocal lesions

## Abstract

**Background:**

Dynamic contrast-enhanced magnetic resonance imaging (DCE-MRI) now has been used to diagnose prostate cancer (PCa). Equivocal lesions are defined as PIRADS category 3 or a Likert scale of 1 to 5 category 3 lesions. Currently, there are no clear recommendations for the management of these lesions. This study aimed to estimate the diagnostic capacity of DCE-MRI for PCa and clinically significant prostate cancer (csPCa) in equivocal lesions.

**Materials and methods:**

Two researchers searched PubMed, Embase and Web of Science to identify studies that met our subject. We searched for articles that mention the accuracy of the diagnosis of DCE-MRI for PCa or csPCa in equivocal lesions and used histopathological results as the reference standard. We used a tool (the Quality Assessment of Diagnostic Accuracy Studies-2 tool) to evaluate the quality of the studies that we screened out. Meta-regression was used to explore the reasons for heterogeneity in results.

**Results:**

Ten articles were eventually included in our study. The sensitivity, specificity and 95% confidence intervals (CI) for DCE-MRI in diagnosing csPCa were 0.67 (95% CI, 0.56–0.76), 0.58 (95% CI, 0.46–0.68). The sensitivity and specificity and 95% CI for DCE-MRI in diagnosing PCa were 0.57 (95% CI, 0.46–0.68), 0.58 (95% CI, 0.45–0.70). The areas under the curve (AUC) of DCE-MRI were 0.67 (95% CI, 0.63–0.71) and 0.60 (95% CI, 0.55–0.64) while diagnosing csPCa and PCa. Through meta-regression, we found that study design, magnetic field strength, the definition of csPCa, and the scoring system were the sources of heterogeneity.

**Conclusion:**

The results of our study indicate that the role of DCE-MRI in equivocal lesions may be limited.

## Introduction

In the causes of cancer-related death for men, prostate cancer (PCa) ranked the fifth globally in 2018 (1). In more than half of the countries, it has been the most frequently diagnosed cancer in men (1). The detection of clinically significant prostate cancer (csPCa) (generally defined as a pathological volume ≥ 0.5 ml or Gleason score ≥ 7) (2, 3) is crucial for lowering the death rate. Because most diagnosed non-clinically significant cancer is a low-grade or indolent lesion which is unlikely to lead to significant mortality (3). Magnetic resonance imaging (MRI) is now an established instrument in diagnosing PCa, with a promising future, and men with elevated prostate-specific antigen (PSA) are increasingly being examined by pre-biopsy MRI (4).

Multiparametric MRI (mpMRI) is a common method for diagnosing prostate cancer, which consists of dynamic contrast-enhanced magnetic resonance imaging (DCE-MRI), diffusion-weighted imaging (DWI), and T2 weighted imaging (T2WI). Currently, whether to include DCE-MRI in a prostate examination is a controversial subject. Biparametric MRI (bpMRI) is defined as consisting of T2WI and DWI without including the sequence of DCE-MRI. Some original articles (5–7) have shown that bpMRI and mpMRI had comparable diagnostic ability in detecting PCa or csPCa, and DCE-MRI has no incremental benefit for improving the recognition of csPCa (8, 9) in equivocal lesions (Prostate Imaging Reporting and Data System category 3 or a Likert scale of 1–5 category 3 lesions) (2, 10). For equivocal lesions, they have the risk of malignancy. In contrast, some studies (11–13) indicated that DCE-MRI had a useful role in upgrading equivocal lesions. DCE-MRI requires intravenous administration of gadolinium contrast agents, which includes additional time and cost, and the safety of the agents that based on gadolinium has been called into question due to some potential toxicities associated with them (e.g., allergy, deposition in brain tissue, and nephrogenic systemic fibrosis) (14). A few conferences dedicated to this topic are being held (for example, European Congress of Radiology 2018) (15). Compared with mpMRI, bpMRI has several advantages without distinctly affecting the shunting action of prebiopsy MRI and detection rate of csPCa (11), such as a noninvasive procedure, avoidance of gadolinium-based contrast agent-related risks, and shorter examination time that might make it more tolerable for people with claustrophobia within the MRI examination and potentially reduce motion artifacts commonly seen with MRI (16).

For prostate cancer examination in MRI, two scoring systems are currently used, the Prostate Imaging Reporting and Data System (PI-RADS) and a Likert scale of 1 to 5. Different imaging methods are using based on the location of the lesions. In PI-RADS v2 (version 2), for lesions in the peripheral zone (PZ), findings on DWI are used as the main results of the score, while for transition zone (TZ) lesions, T2WI is the primary component of the score (17). When T2W imaging and DWI have diagnostic quality, DCE-MRI plays a minor role in determining the category of PIRADS; for example, a positive DCE-MRI outcome might upgrade the lesion of PIRADS 3 (equivocal lesions) to PIRADS 4 in PZ (17). PI-RADS v2.1 clarifies some ambiguities and discrepancies of v2 and some technical aspects have been updated, but the role of DCE-MRI remains unchanged (18). Based on the current evidence, the equivocal lesion on behalf of a “gray zone” and does not provide clear recommendations (10).

At present, most of the previous articles have compared the diagnostic efficiency of bpMRI and mpMRI, in our study, we directly focused on the diagnostic efficiency of DCE-MRI for csPCa and PCa in equivocal lesions. Whether DCE-MRI could upgrade category 3 lesions (equivocal lesions) to category 4 remains to be discussed. To determine the diagnostic ability of DCE-MRI in such lesions, we collected studies that reported lesions with a score of 3 on BPMRI (or on DWI and T2WI), and for these lesions, mpMRI or DCE-MRI was also applied. The endpoint was that the patients with category 3 lesions were upgraded to category 4 in prostate cancer scoring systems such as PI-RADS and Likert scale due to positive DCE findings.

## Materials and Methods

### Literature Search

According to PICOS criteria (19) (patient populations, interventions, comparators, outcomes, and study design), we developed the following study question for our meta-analysis: What is the diagnostic performance of DCE-MRI in diagnosing both PCa and csPCa in equivocal lesions, with pathological results as a reference standard?

PubMed, Embase, and Web of Science were systematically reviewed by two independent researchers to identify studies that mentioned or calculated the diagnostic accuracy of DCE-MRI in diagnosing PCa or csPCa in equivocal lesions that scored 3 points in PI-RADS or a Likert scale of 1 to 5. The strategy was as follows: ((((biparametric OR bpMRI OR ((T2WI OR T2 weighted MRI) AND (DWI OR diffusion-weighted MRI)))) AND (multiparametric OR mpMRI OR ((T2WI OR T2 weighted MRI) AND (DWI OR diffusion-weighted MRI) AND (DCE-MRI OR dynamic contrast-enhanced magnetic resonance imaging)))) AND (MRI OR magnetic resonance imaging)). The search was performed on December 17, 2020, without a start date limit. We only screened studies written in English or Chinese, and also checked the references of the relevant articles to find other studies that meet the criteria.

Originally, two readers screened the titles and abstracts independently, and the final analysis included articles that were carefully chosen after the full texts were read. Discrepancies were solved by consensus. When a consensus cannot be reached, a third reader was invited to resolved disagreements.

### Study Selection

Retrospective and prospective studies that reported equivocal lesions (PIRADS 3 OR Likert score 3) that identified by prostate MRI were included. All the cases had been diagnosed pathologically. In addition to DCE-MRI, other MRI sequences in the studies included T2WI and DWI. Studies in which only compared DWI with DWI+DCE-MRI outcomes were excluded. Excluding studies whose data were insufficient to create a 2 × 2 contingency table.

### Patients

The subjects of the study included the following: (1) men with an abnormal digital rectal examination (DRE) or clinically suspected of having PCa for elevated prostate-specific antigen (PSA); and (2) patients that had neither surgery nor chemotherapy before MRI examination. Recurrent PCa patients were excluded. We also excluded the patients who did not meet the subject of the study. The main observational indexes of this study were the detection of csPCa and PCa, independent of where the lesions were located.

### Data Extraction and Quality Assessment

The information extracted from the studies were as follows: (1) characteristics of study: the authors’ name, publication year, country, study design (prospective, retrospective, or no report), the definition of csPCa; (2) patient characteristics: patient number, patient age, biopsy-naïve (yes or no), location of lesions, PSA levels, prostate volume, prostate-specific antigen density (PSAD); and (3) imaging characteristics: coil, the b values of DWI sequence, magnetic field strength, and the description of the reference standard. When multiple readers provided each result independently, we used the average value because the interobserver agreement of studies included was generally favorable. True and false positive and true and false negative (TP, FP, TN, and FN, respectively) from included studies were extracted to compute sensitivity, specificity, positive likelihood ratio (PLR), negative likelihood ratio (NLR), diagnostic odds ratios (DOR) and area under the curves (AUC).

We applied the revised instrument for the Quality Assessment of Diagnostic Accuracy Studies tool (QUADAS-2) to assess the quality of the included studies (20) by RevMan software (version 5.3). The risks of bias were also scored, such as patient selection and index texts.

### Statistical Analysis

The coupled forest plots of the sensitivity and specificity were drawn. To summarize the diagnostic accuracy, we also drew the summary receiver operating characteristic curve (SROC) to calculate AUC (AUC range of 0.5–1.).

The inconsistency index (I^2^ value) and Cochran’s Q test were used to test the existence of heterogeneity, with I^2^> 50% or P< 0.05 suggesting considerable heterogeneity was present. Based on the value of I^2^, different models were used: a random-effects model if I^2^ > 50%, and a fixed-effect model if I^2^ < 50%. If obvious heterogeneity was noted, sensitivity analysis would be performed. For figuring out the heterogeneity of the 10 studies, meta-regression analysis was also conducted: study design (retrospective or prospective), the definition of csPCa, magnetic field strength (3-T, 1.5-T, or both), providing individual results of multiple independent readers (yes or not), the location of lesions and the scoring system (PIRADS or a Likert scale of 1–5). Publication bias was analyzed by Deek’s Funnel plot. All statistical analyses were carried out by using Stata software (version 12.0, StataCorp).

## Results

### Literature Search

**Figure 1:**
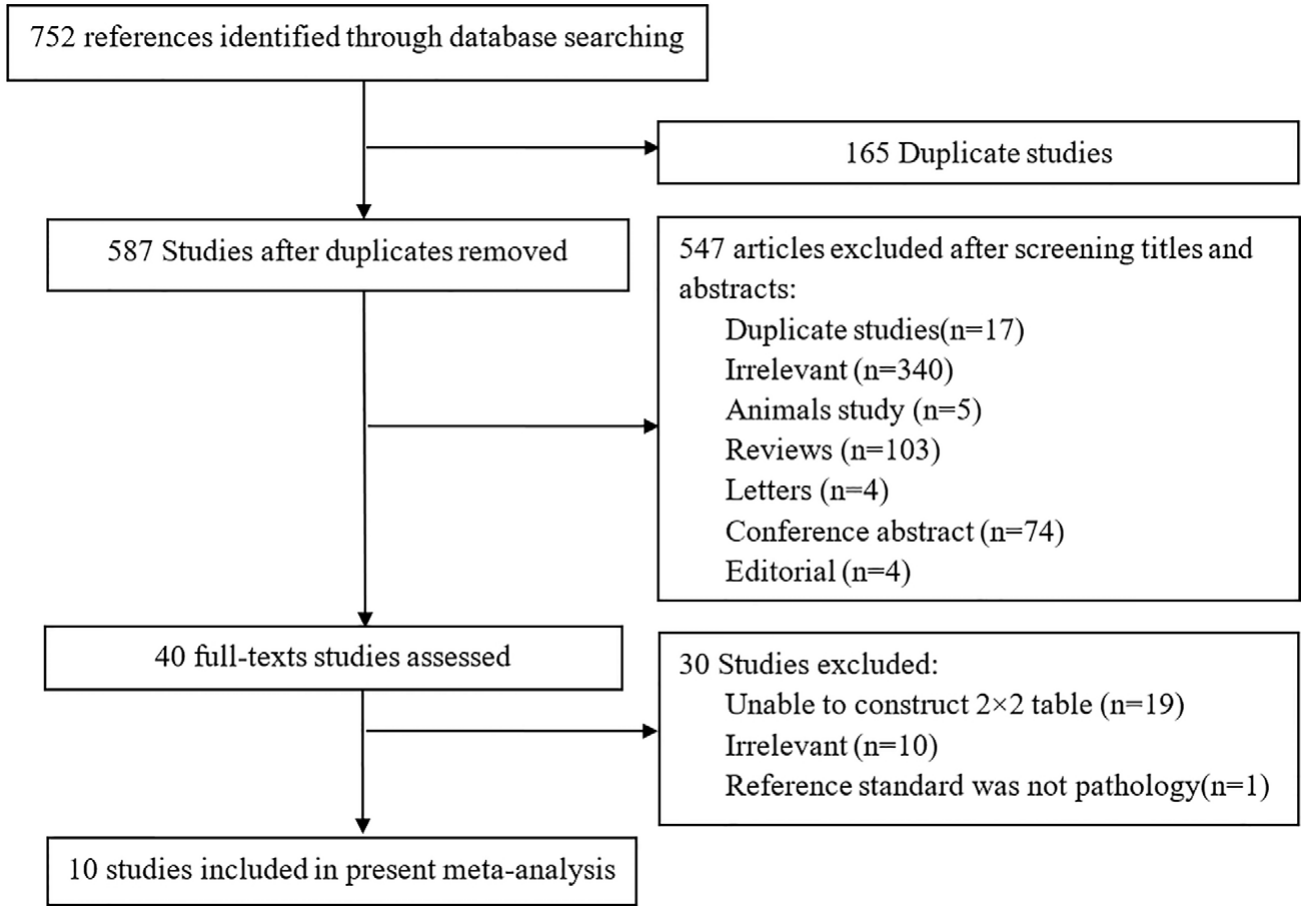
Flowchart of study selection for present meta-analysis.

In searching the PubMed, Embase and Web of Science, a total of 752 studies were obtained, and 165/752 studies were repetitive. After reviewing the titles and abstracts, 547 studies were excluded. Forty studies were considered to be possibly related to our meta-analysis, and the full-texts of the studies were read in detail. A total of 10 studies including 752 patients and 831 lesions were ultimately included in the present analysis (8, 9, 21–28) to calculate the diagnostic ability of DCE-MRI for csPCa, while five of the 10 studies were used to assess the capacity of DCE-MRI in diagnosing PCa (8, 21, 23, 24, 26). Because Druskin et al. did not report the specific numbers of PIRADS 3 patients (21), we estimate the number of patients in this group (PIRADS 3) to be 213 based on the information given in the study. **Figure 1** displays an overview of the search process.

### Study Characteristics

**Table 1** presents the characteristics of the study and patients of the included studies. The countries of the 10 studies are as follows: Three studies were from The United Kingdom, Belgium and Korea, respectively. Three studies were conducted in the United States of America and four in China. Nine of 10 studies were conducted retrospectively and one was prospective. The ages of the included patients were from 30 to 87 years old, and the ranges of the mean or median PSA values were 0.22 to 935.5 ng/ml. Four studies reported the prostate volume, ranging from 8.5 to 206 ml. PSAD of the patients were only reported by three studies, ranging from 0.002 to 3.34 ng/ml/ml. The locations of the lesions of included studies were as follows: 4 in PZ (peripheral zone), 4 at the PZ and TZ (transitional zone), and 3 studies did not report the location of lesions. Among 10 studies, one study used 1.5-T MRI, 8 studies used an MRI machine with a magnetic field strength of 3-T, and the remaining one applied 1.5-T and 3-T MRI. Further, Taghipour et al. (27) and some patients of Druskin et al. (21) used endorectal coil. Two studies used pure radical prostatectomy as the reference standard. Systematic biopsies, the transperineal prostate mapping (TPM) biopsy, MRI/ultrasound (US) fusion-targeted biopsy with transperineum systematic biopsy, or transrectal ultrasonography (TRUS)-guided biopsy were applied as the reference standards in eight studies. Nine of ten studies used PIRADS as a scoring system and one used a five Likert scale of 1 to 5. The technical features of the ten studies are summarized in **Table 2**.

**Table 1:** Characteristics of studies included in the meta-analysis.

First Author (Year of Publication	Country	Study design	No. of Patients	Biopsy-naïve	Location of Lesions	No. of lesions	age(y)^a^	PSA (ng/ml)^a^	Prostate Volume (ml)^a^	PASD (ng/ml/ml)^a^	Definition of csPCa	Scoring System
Han et al. (22)	China	Retrospective	13	NR	NR	13	66.3 (42.0–83.0)	7.23 (4.15–10, 00)	NR	0.21 (0.05–0.90)	Gleason score≥ 7	PI-RADS v2.1
Wang et al. (8)	China	Retrospective	75	Yes	PZ	75	69 (47–84)	8.11 (4.06–123.00)	42.4 (8.5–107.8)	0.20 (0.06–3.34)	Gleason Score≥7	PIRADS v2
Xu et al. (23)	China	Retrospective	29	Yes	NR	29	66.87 (58.34–75.4)	4.65 (0.22–86.00)	NR	NR	Gleason score≥7, volume greater than 0.5 cm3, extra-prostatic extension	PIRADS v2
Zhang et al. (24)	China	Retrospective	81	Yes	PZ+TZ	81	70.89 (62.42–79.36)	13.00 (4.83–94.58)	NR	NR	Gleason score≥7	PIRADS v2
Choi et al. (9)	Korea	Retrospective	29	No	PZ+TZ	29	65 (58–72)	7.90 (0.90–14.90)	NR	NR	Gleason score≥7, volume greater than 0.5 cm3, extra-prostatic extension	PIRADS v2
Roh et al. (26)	USA	Retrospective	62	NR	PZ+TZ	69	66 (30–87)	7.60(5.40–11.00)	60 (16.0–206.0)	0.17 (0.01–1.05)	Gleason scores ≥7	PIRADS v2
Taghipour et al. (27)	USA	Retrospective	45	NR	PZ	45	59 (53–65)	6.70 (2.00–11.40)	NR	NR	Gleason scores ≥7	PIRADS v2
De Visschere et al. (25)	Belgium	Retrospective	47	NR	PZ	47	66 (44–85)	9.00(1.40–935.50)	49 (19.8–201.0)	NR	Gleason score≥7, volume greater than 0.5 cm3, extra-prostatic extension	PIRADS v2
Druskin et al. (21)	USA	Retrospective	213	some	PZ	285	63.6 (58.2–68.7)	5.90 (4.20–8.60)	51.6 (34.0–73.0)	NR	Gleason scores ≥7	PIRADS v2
Bosaily et al. (28)	UK	Prospective	158	Yes	NR	158	64 (58–69)	6.50 (5.00–8.80)	NR	NR	Gleason score≥4 + 3 or cancer core length 6 mm of any grade	a Likert score of 1–5

a Data are medians or means with range in paratheses.

PSA, prostate-specific antigen; PSAD, prostate-specific antigen density; csPCa, clinically significant prostate cancer; USA, the United States of America; UK, The United Kingdom; NR, not reported; PZ, peripheral zone; TZ, transition zone.

**Table 2:** Technical characteristics of studies included in this meta-analysis.

First Author (Year of Publication)	Magnetic Field Strength (T)	Coil	b Values (s/mm^2^)	Readers and Years of Experience	Reference Standard (Methods)	Interval between MRI and Reference Standard (days)	Pathologists	Blinding to Pathological Results
Han et al. (22)	3	Phased-array coil	800, 1,000, 1,200, 1,400	2 Radiologists with more than 5 years of experience with the PI-RADS	TRUS-guided biopsy	NR	NR	YES
Wang et al. (8)	3	Pelvic mpMRI 16-channel phased array coil	0, 800, 1,500	2 Dedicated radiologists over 10 years of prostate mpMRI experience	TB and SB	NR	2 Genitourinary pathologists	YES
Xu et al. (23)	3	Abdominal eight-channel surface phased array coil	100, 150, 200, 500, 800, 1,000, 1,500, 2,000	2 Radiologists with 5 and 13 years of experience	Prostatic biopsy or prostatectomy	NR	NR	YES
Zhang et al. (24)	3	Body-phased array coil	0, 100, 1,000, 2,000	2 Radiologists with more than 3 and 7 years of experience	TRUS-guided biopsy; TB; TURP, prostatectomy		Expert uropathologists	YES
Choi et al. (9)	3	NR	Institution 1 = 0, 1,000 or 0, 100, 1,000 Institution 2 = 0, 50, 500, 1,000	2 Radiologists with 7 and 13 years of experience	Radical prostatectomy	25.2 ± 13.9	Expert pathologist	YES
Roh et al. (26)	3	NR	50, 800, 1,200	13 Fellowship-trained body radiologists with experience ranging from 6 to 38 years	14-core systematic biopsies and TB	NR	NR	NR
Taghipour et al. (27)	3	Endorectal coil	0, 500, 1400	1 radiologist with 14 years of experience in prostate MRI	Radical prostatectomy	≥ 50	NR	YES
De Visschere et al. (25)	3	None endorectal coil	50, 250, 500, 750, 1000	NR	A systematic 12-core TRUS-guided prostate biopsy	NR	NR	NR
Druskin et al. (21)	1.5, 3	Endorectal coil (some patients)	NR	Radiologists	MRI TRUS-fusion TB and SB	NR	Urologists	NR
Bosaily et al. (28)	1.5	Pelvic-phased array coil	0, 150,500, 1000, 1400	Radiologists from the 11 UK centers in the trial had experience of reporting MP-MRI	The transperineal prostate mapping biopsy	NR	2 Expert uropathologists	YES

NR, not reported; mpMRI, multiparametric magnetic resonance imaging; UK, The United Kingdom; TRUS, transrectal ultrasonography; TB, MRI/ultrasound fusion-targeted biopsy; SB, transperineum systematic biopsy; TURP, transurethral resection of the prostate.

### Quality Assessment

**Figure 2:**
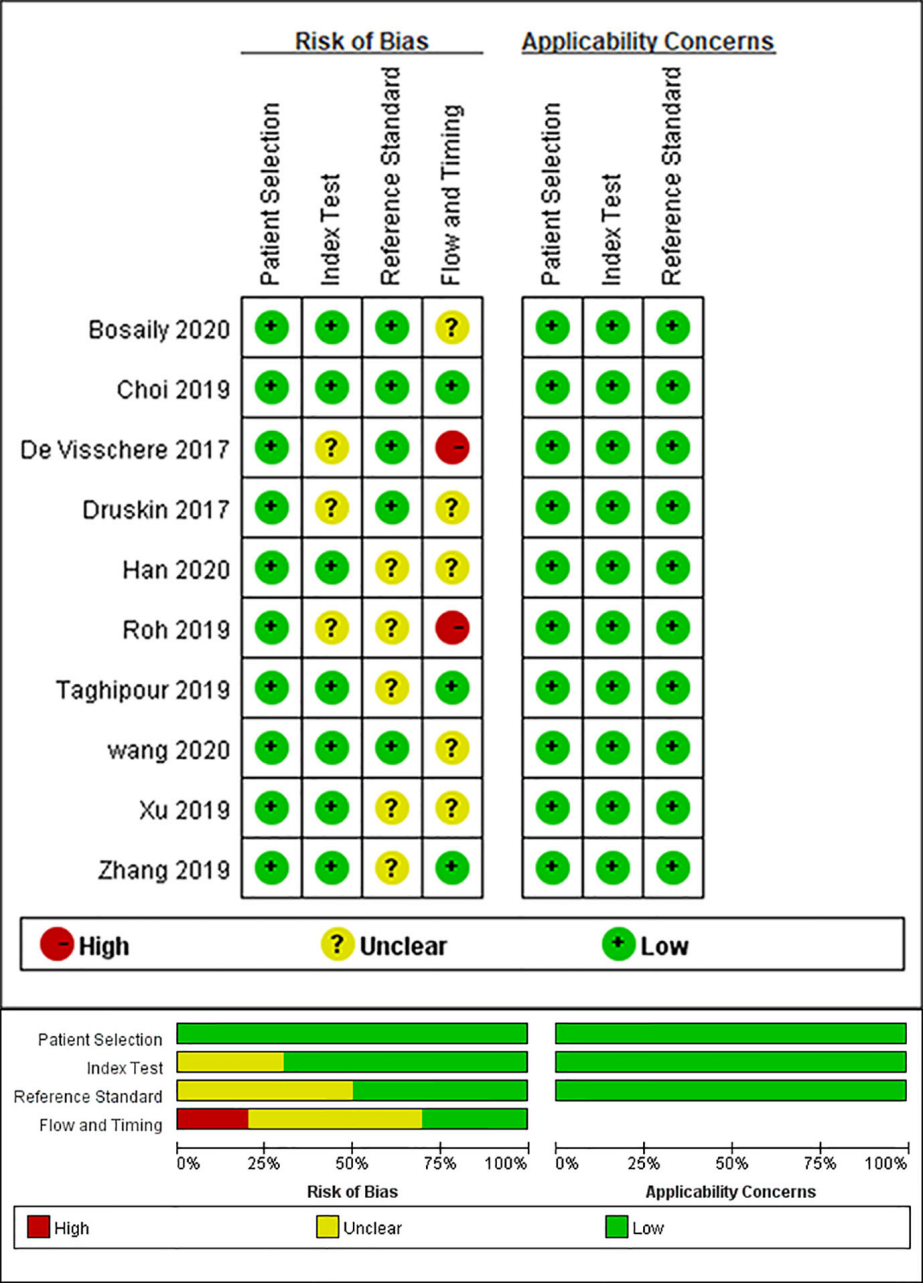
Grouped bar chart shows risk of bias (left) and concerns for applicability (right) for included studies according to revised Quality Assessment of Diagnostic Accuracy Studies tool (QUADAS-2).

The main causes of bias include reference standard, index test, and flow and timing. **Figure 2** shows the results of the QUADAS-2 assessment. Three studies did not report the blinding of the interpretation of DCE-MRI, and three studies did not specify whether the results of the pathology were explained without the knowledge of the DCE-MRI. The bias of flow and timing was caused by, in some studies, an unknown interval between DCE-MRI and pathology tests, and not all patients of one study were included in this analysis (because the remaining patients did not meet the subject of this study). There were no concerns about the capability of qualified studies to answer the research questions.

### Data Analysis

**Figure 3:**
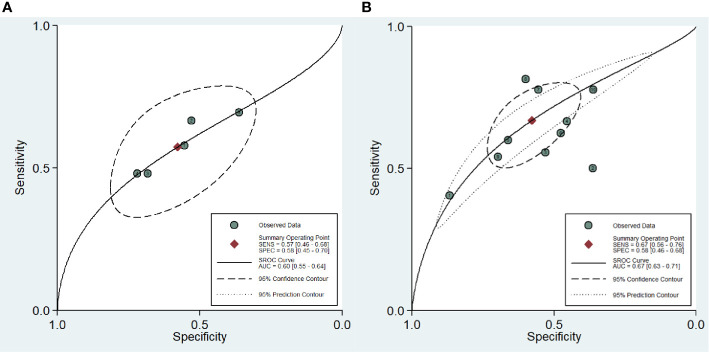
Summary receiver operating characteristic curve (SROC) of the diagnostic performance of DCE-MRI in detecting prostate cancer **(A)** and clinically significant prostate cancer **(B)**.

For the ten studies, we found that the pooled sensitivity, specificity and 95% confidence intervals (CI) for DCE-MRI in diagnosing csPCa and PCa were low—0.67 (0.56–0.76), 0.58 (0.46–0.68), 0.57 (0.46, 0.68), 0.58 (0.45, 0.70), respectively. The PLR, NLR and AUC for DCE-MRI in diagnosing csPCa and PCa were 1.6, 0.57, 0.67 and 1.4, 0.74, 0.60, respectively, suggesting that DCE-MRI may not be necessary in diagnosis. These data are summarized in **Table 3**, and the SROC plots with 95% CI area are shown in **Figure 3**.

**Table 3:** Summary performance of DCE-MRI for the diagnosis of csPCa and PCa.

	Analysis (No. of Studies)	
	The diagnostic ability of DCE-MRI for csPCa in equivocal lesions (10)	The diagnostic ability of DCE-MRI for PCa in equivocal lesions (5)
Sensitivity	0.67 (95% CI, 0.56–0.76)	0.57 (0.46, 0.68)
Specificity	0.58 (95% CI, 0.46–0.68)	0.58 (0.45, 0.70)
PLR	1.6 (95% CI, 1.3–2.0)	1.4 (1.1, 1.7)
NLR	0.57 (95% CI, 0.45–0.73)	0.74 (0.59, 0.93)
DOR	3 (95% CI, 2–4)	2 (1, 3)
AUC	0.67 (95% CI, 0.63–0.71)	0.60 (0.55–0.64)

PCa, prostate cancer; csPCa, clinically significant prostate cancer; PLR, positive likelihood ratio; NLR, negative likelihood ratio; DOR, diagnostic odds ratios; AUC, the area under the summary receiver operating characteristic curves; CI, confidence intervals.

**Figure 4:**
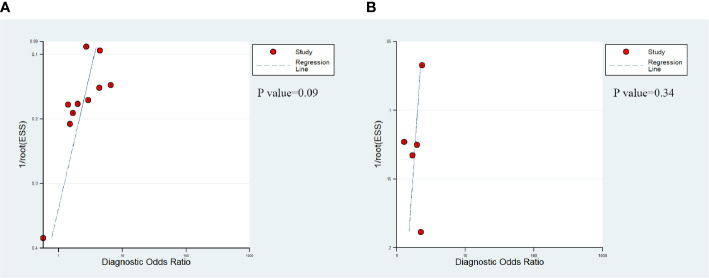
Deek's funnel shows that publication bias may be present for DCE-MRI in detecting clinically significant prostate cancer (P=0.09) **(A)** and the likelihood of publication bias is low for DCE-MRI in detecting prostate cancer (P=0.34) **(B)**.

The presence of publication bias of DCE-MRI in csPCa detection was found by a funnel plot asymmetry test in Deeks et al. (29); no publication bias was noticed in PCa detection, as shown in **Figure 4**.

### Exploration of Heterogeneity

**Figure 5:**
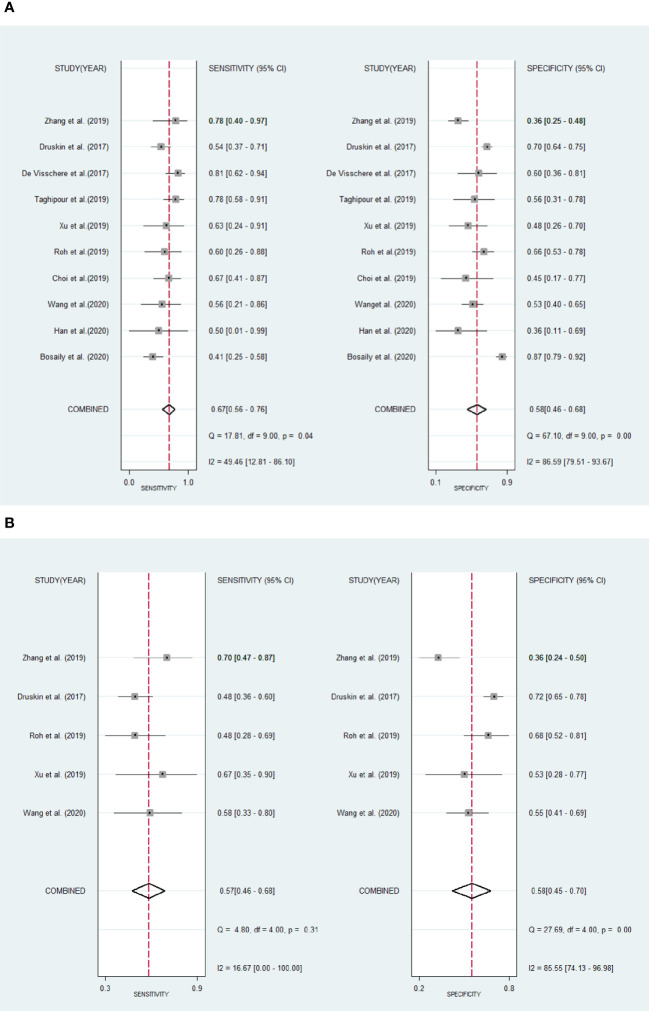
Forest plots show pool sensitivity and specificity of DCE-MRI in the diagnosis of clinically significant prostate cancer **(A)** and prostate cancer **(B)**. Horizontal bar indicates 95% confidence intervals of individual studies. Diamond shows combined results.

The existence of heterogeneity was found by the Cochran’s Q test (Q=35.354, p=0.001). The forest plots (**Figure 5**) also shown heterogeneities in the detection of csPCa (I^2^ of sensitivity and specificity were 16.67%, 85.55%, respectively), and PCa (I^2^ of sensitivity and specificity were 49.46%, 86.59%, respectively) in DCE-MRI.

**Table 4** shows the results of the meta-regression analysis. Study design, magnetic field strength, the definition of csPCa and the scoring system were identified as the factors that lead to heterogeneity. Further, sensitivity analysis showed that the results of DCE-MRI in diagnosing csPCa are relatively robust.

**Table 4:** Results of meta-regression analysis.

Parameter	Category (No. of Studies)	Sensitivity (%)	Specificity (%)	LRT Chi-Square	P value (Joint Model)
Study design	Retrospective (9) Prospective (1)	0.69 (0.61–0.78) 0.40 (0.20–0.61)	0.54 (0.46–0.62) 0.87 (0.77–0.97)	8.74	0.01
The definition of csPCa	Gleason Score≥7 (5) Gleason score≥4 + 3 or cancer core length 6 mm of any grade (1) Gleason score ≥ 7 or a volume ≥ 0.5 cm3 or extra-prostatic invasion (4)	0.69 (0.56–0.83) 0.40 (0.20–0.61) 0.71 (0.57–0.86)	0.51 (0.37–0.66) 0.87 (0.77–0.97) 0.56 (0.38–0.75)	1.97 8.74 0.54	0.37 0.01 0.77
Magnetic field strength	1.5-T (1) 3-T (8) Both (1)	0.40 (0.20–0.61) 0.72 (0.63–0.81) 0.54 (0.27–0.81)	0.87 (0.77–0.97) 0.51 (0.41–0.60) 0.56 (0.45–0.68)	8.74 10.02 1.04	0.01 0.01 0.60
Providing individual results of multiple independent readers	Yes (1) No (9)	0.67 (0.37–0.97) 0.67 (0.56–0.78)	0.45 (0.02–0.88) 0.59 (0.47–0.70)	0.46	0.79
Location	PZ (5) PZ+TZ (3) NR (2)	0.70 (0.58–0.83) 0.68 (0.49–0.87) 0.52 (0.31–0.73)	0.55 (0.39–0.71) 0.50 (0.30–0.70) 0.74 (0.57–0.91)	0.65 0.94 3.13	0.72 0.62 0.21
Scoring system	PIRADS (9) a Likert scale of 1–5 (1)	0.69 (0.61–0.78) 0.40 (0.20–0.61)	0.54 (0.46–0.62) 0.87 (0.77–0.97)	8.74	0.01

%, 95% confidence intervals; LRT, likelihood-ratio test; csPCa, clinically significant prostate cancer.

## Discussion

Several published meta-analyses (15, 30, 31) that have compared the diagnostic efficiency of bpMRI and mpMRI found that bpMRI offers comparable detecting performance as mpMRI while diagnosing prostate cancer. However, few of them mention the diagnostic performance of DCE-MRI in equivocal lesions. In our study, we wanted to know if DEC-MRI has a predictive value for PCa and csPCa in equivocal lesions. Based on that, our meta-analysis included 10 studies to investigate the role of DCE-MRI in such lesions. Our study bridges this lacking information and supports this meta-analysis by finding that the benefit of DCE-MRI in the diagnosis of equivocal lesions is limited.

The AUC (0.60) of DCE-MRI in diagnosing PCa in our study is low. A study led by Vargas et al. (3) which used PI-RADSv2 as a scoring system, DCE-MRI only helped to detect four out of 152 tumors. A study conducted by Xu et al. (23) found that PCa in equivocal lesions may not have an early enhancement in DCE-MRI and no significant difference was noted between positive or negative DCE-MRI findings.

The results (sensitivity, specificity, and AUC) of DCE-MRI in detecting csPCa in equivocal lesions were also shown to be not so satisfactory. Choi et al. (9) in a 2019 study observed the csPCa detection rate of DCE-MRI in equivocal lesions. The results of them are similar to ours. In their study, positive DCE-MRI results (csPCa was detected) were slightly higher than that of bpMRI, but the difference was not statistically significant. Wang et al. (8) found that only 5 cases of csPCa were detected by DCE-MRI in 75 equivocal lesions. There was no distinct difference in the identification of csPCa between positive DCE-MRI lesions and negative DCE-MRI lesions. In biopsy-naïve patients with category 3 lesions, whether the DCE-MRI outcome is positive or negative, the risk of csPCa in them is similar. It might indicate that DCE-MRI can be omitted without sacrificing risk stratification in that group of patients (8, 21).

The possible reasons for the unsatisfied diagnostic accuracy and specificity of DCE-MRI in equivocal lesions are as follows. The large overlapping of benign prostate hyperplasia (BPH) and the enhancement patterns of cancer may have led to false-positive results (32, 33). Further, among men with prior negative systematic prostate biopsy (PNB), there may be more false-positive mpMRI results. Because post-biopsy hemorrhage can affect the interpretation of images (34), and post-biopsy prostatitis may cause false-positive results in mpMRI (prostatitis, a known complication of prostate biopsy) (21). In addition, the minimal additional benefit of DCE-MRI might be offset by its use-related disadvantages, such as additional cost and scan time, as well as the danger of side effects from contrast media (3).

Some methods have been proposed to help improve the diagnostic performance of prostate MRI (without DCE-MRI) in equivocal lesions. One study noticed that when combined with PSAD, ADC_mean_ (the mean apparent diffusion coefficient value, derived from DWI) and age, it has the greatest net benefit and higher csPCa detection rate than any single method (age, ADC_mean_, or PSAD) (8). Thus, among equivocal lesions, clinical parameters may better help identify csPCa. Based on a retrospective study by Sheridan et al., three clinical parameters were determined to be useful in predicting csPCa among PI-RADS 3 lesions, including an abnormal digital rectal examination (DRE) finding, age≥ 70 years, and gland size ≤ 36 ml (2). High negative predictive value (NPV) and specificity could be seen while incorporating any of the two above-mentioned clinical parameters with a PI-RADS 3 finding (2). Their findings may be valuable in determining whether future biopsies are needed for PI-RADS 3 lesions (2).

One essential aspect of any meta-analysis is exploring heterogeneity. In the present meta-analysis, moderate to high heterogeneity was detected among these studies. Based on meta-regression analysis, the factors, namely, study design, magnetic field strength, the definition of csPCa, and scoring system all affect heterogeneity. We found a study (28) that had all the above factors. We conducted a sensitivity analysis based on the study to estimate whether the results might be markedly affected by the study. After excluding this study, the sensitivity and specificity of DCE-MRI in diagnosing csPCa were 0.69 (0.61, 0.78) and 0.54 (0.46, 0.62), respectively. We found that the results of our study are robust after comparing these values with the results (the sensitivity and specificity of the original 10 studies).

Magnetic field strength is one of the sources of heterogeneity. Due to its high image quality, 3-T MRI is widely used for prostate exams. 3-T MRI has a higher signal-noise ratio (SNR), which improves the ability to detect smaller lesions compared to 1.5-T MRI.

The definition of csPCa in the 10 included studies is mainly divided into three types. The first is Gleason Score≥7, the second is Gleason score 4 + 3 or cancer core length 6 mm of any grade, and the third is Gleason Score≥7 or Gleason score ≥ 7 and/or a volume ≥ 0.5 cm3 and/or extra-prostatic invasion. The third definition covers the broadest range of lesions, and according to our results of meta-regression analysis, we found that it also has the highest sensitivity. It indicated that when a looser definition is used, is likely to increase the detection rate of small tumor foci (32). The different definition of clinically significant PCa introduces heterogeneity, thus the result of our meta-analysis should be applied with caution.

In these studies of our meta-analysis, PI-RADS v2 or PI-RADS v2.1 or a Likert scale of 1 to 5 was adopted to assign the possibilities of PCa. We found different scoring systems, such as PI-RADS and a Likert scale of 1 to 5, are some of the sources of heterogeneity. Likert score is subjective scoring and PI-RADS is semi-objective scoring. It is reported that semi-objective scoring might be easier for less experienced radiologists because it provides a framework on which inexperienced readers could reference while interpreting the image, while subjective scoring requires experience (3, 35).

Several limitations can also be seen in this meta-analysis. First, in the scoring system of PI-RADS, DCE-MRI was mainly applied in the lesions of category 3 PZ lesions, yet, category 3 lesions in three studies were located both in PZ and TZ, and the specific locations of lesions were not reported in two studies. A subgroup analysis on the location of lesions was omitted because this information was limited in the studies analyzed. Second, the risk of bias of index test for some included studies was unclear because three studies did not report whether the interpretation of DCE-MRI was blinded to the knowledge of reference standard. It is not clear if this unclear risk will affect the outcomes. Third, the diagnostic capability computed in this meta-analysis might not be widely applied in all MRI readers. Among relatively experienced readers, the inter-reader reproducibility tends to be higher than that among less experienced readers (36), however, when some studies offered the results of each interpreter with different experiences, we used the mean value. Fourth, publication bias was found when exploring the diagnostic performance of DCE-MRI to csPCa, thus, more studies are needed to verify the outcome. According to the sensitivity analysis at this meta-analysis, we found that the outcomes are relatively stable. The conclusion of our meta-analysis is not applicable for the detection of recurrence of prostate cancer after treatment.

## Conclusion

The available results might suggest that DCE-MRI provides no incremental benefit in the detection of csPCa and PCa in equivocal lesions. Because of the poor performance of DCE-MRI, more research is needed to find more suitable methods to improve the detection rate of csPCa and PCa in equivocal lesions.

## Data Availability Statement

The original contributions presented in the study are included in the article/supplementary materials. Further inquiries can be directed to the corresponding authors.

## Author Contributions

JZ and QC contributed to the collection of data for the study. DZ and MF contributed to the analysis and interpretation of data. JZ wrote the draft of the manuscript. QC contributed to the language revising and reviewed the manuscript. CS and LL contributed to the conception and design of this meta-analysis. All authors contributed to the article and approved the submitted version.

## Funding

This work was supported by the National Natural Science Foundation of China (Grant Number: 21317241, 81971672) and the Fundamental Research Funds for the Central Universities (Grant Number: 21620308, 21620101).

## Conflict of Interest

The authors declare that the research was conducted in the absence of any commercial or financial relationships that could be construed as a potential conflict of interest.
